# Impact of COVID-19 on individual mental health and maternal health services in Ethiopia: systematic review and meta-analysis

**DOI:** 10.3389/fpubh.2024.1407269

**Published:** 2024-06-24

**Authors:** Melsew Setegn Alie, Desalegn Girma, Yilkal Negesse, Amanuel Adugna, Gossa Fetene Abebe

**Affiliations:** ^1^Department of Public Health, School of Public Health, College of Medicine and Health Science, Mizan-Tepi University, Mizan-Aman, Ethiopia; ^2^Department of Midwifery, College of Medicine and Health Science, Mizan-Tepi University, Mizan-Aman, Ethiopia; ^3^Department of Public Health, College of Medicine and Health Science, Debre Markos University, Debre Markos, Gojjam, Ethiopia

**Keywords:** SARS, COVID-19, COVID, SARSCoV-2, pandemic

## Abstract

**Background:**

The COVID-19 pandemic has caused a major outbreak in the 21st century and has led to significant mental health hazards worldwide. To address this issue, a systematic review has been conducted to analyze existing literature on the impact of COVID-19 on the psychological well-being of the general population, as well as the associated risk factors.

**Methods:**

A comprehensive search was carried out on PubMed, Embase, Medline, Web of Science, and Scopus databases, covering all available literature up until February 20, 2024. This search was conducted in accordance with the PRISMA guidelines, ensuring a systematic approach. The selection of articles was based on predetermined eligibility criteria, ensuring the inclusion of appropriate and suitable research. In the final analysis, a total of 15 articles focusing on depression and anxiety, 11 articles on stress, and 7 articles on psychological problems were included. These articles specifically examined the outcome variables within the context of English language and specific areas. For the meta-analysis on maternal health services, 11 articles were included for family planning, 25 articles for postnatal care services, 16 articles for institutional delivery, and 14 articles for safe abortion services. These articles were carefully selected for the final pooled analysis.

**Result:**

According to a recent systematic review, anxiety, depression, stress, and psychological distress have been prevalent in Ethiopia during the COVID-19 pandemic, with rates of 40, 41, 23, and 41%, respectively. The review also identified various sociodemographic factors that have impacted the country’s response to the pandemic, including female gender, age, marital status, incarceration, low income, and lack of social support. Furthermore, the review found that maternal health services have experienced significant reductions during the pandemic.

**Conclusion:**

The COVID-19 pandemic has led to a significant increase in psychological distress, which in some cases, is severe enough to require clinical treatment. It is crucial to prioritize efforts to address the negative impact of COVID-19 on mental health as a global public health priority. Additionally, it is important to pay attention to maternal health services during COVID-19 mitigation programs.

## Introduction

The COVID-19 pandemic has had significant negative impacts on global healthcare systems, the world economy, and societal structures (1, 2). Measures such as nationwide lockdowns and fear of seeking healthcare have disrupted services, potentially affecting the well-being of mothers and their babies (3, 4). Maternal and child health services are facing considerable challenges as a result of the pandemic (5, 6). A study conducted in low and middle-income countries estimated that a modest decline of 10% in essential maternal and newborn healthcare coverage could lead to 28,000 maternal deaths due to the pandemic (7). This reduction in services, coupled with changes in healthcare-seeking behavior, has been linked to worsened maternal health outcomes, including an increase in maternal deaths, ectopic pregnancies, maternal depression, ruptured pregnancies, and stillbirths (8). Additionally, there is emerging evidence suggesting that fetal outcomes have also worsened, with higher rates of preterm birth and stillbirth observed during the COVID-19 pandemic (9, 10). The pandemic has therefore posed significant challenges to maternal and child health globally.

The COVID-19 pandemic has posed significant challenges for many countries in maintaining essential maternal, newborn, and child health services. Women may face difficulties accessing maternal healthcare due to issues such as transportation problems, restrictions, anxiety, and fear of potential exposure to the coronavirus (6, 11). A systematic review and meta-analysis have shown a significant decrease in the utilization of essential maternal healthcare services (12, 13). The diversion of resources towards the pandemic response has led to the disruption of basic maternal, newborn, and child health services, increasing the risks of maternal illness and death (14). This situation presents a further challenge in providing essential services throughout the entire maternity continuum of care while simultaneously dealing with COVID-19 (15). Recent evidence suggests that government measures implemented to combat COVID-19, such as stay-at-home guidance, women’s healthcare-seeking behavior, community perception, perceived poor quality of care during the pandemic, and fear of contracting the virus, have all influenced the maternity care provided to mothers during pregnancy, childbirth, and the postpartum period (11, 16). Ethiopia reported its first confirmed case of COVID-19 on March 13, 2020. Following the declaration of the pandemic, local mobility was restricted in Ethiopia, gatherings in all settings were prohibited, and individuals suspected of having acquired the virus were advised to report to the nearest health authority (17, 18). The ongoing pandemic has resulted in a range of mental and psychological problems for individuals, which can have negative effects on their self-care practices, appetite, sleep, immunity, and ability to follow healthcare guidelines. Anxiety, depression, panic attacks, and even psychotic symptoms have been frequently reported. These impacts have been particularly high among healthcare professionals and communities (19, 20). Despite the high costs associated with neglecting the mental health impact of disease outbreaks, it is often overlooked during pandemic management. Early evidence suggests that healthcare workers directly involved in treating COVID-19 patients are at risk of developing mental health symptoms (20). In Ethiopia, some research’s (21–23) were conducted to assess the impact of COVID-19 while the previous one never included the current impact and most were done immediately after the COVID-19 pandemic declared. The COVID-19 pandemic has created gaps in our knowledge about the impact on mental health and maternal health service. Understanding how lockdown measures affect maternal health services and mental health is crucial. Despite extensive literature on impact of COVID-19 on mental health during COVID-19, there is no systematic review focusing solely on perinatal women’s experiences and mental health during lockdown and after the lockdown. This understanding is essential for better support during and after the pandemic. We need to comprehend the effects of restrictions on women’s perinatal mental health to improve best practices in supporting them. To mitigate the maternal and mental health service after the pandemic pooled and synthesized evidences were highly crucial. In addition the health impact of COVID-19 in Ethiopia never synthesize with the current evidences. In Ethiopia, there is a lack of nationwide evidence on the impact of COVID-19 on individuals and healthcare services. Existing studies are fragmented and provide varying reports. To address this gap, a systematic review and meta-analysis were conducted to estimate the overall impact of COVID-19 on the health of individuals and healthcare services in Ethiopia.

## Methods

### Research questions

To conduct a systematic review on the pooled prevalence of long-term effects of COVID-19 in Ethiopia, we structured the research question using the PICO (S) format. The participants (P) included individuals who had been clinically diagnosed or laboratory-confirmed to have COVID-19. The intervention (I) and comparison (C) in this systematic review and meta-analysis involved individuals with COVID-19 impact and those without COVID-19 impact, respectively. The outcome (O) of interest was the impact of COVID-19. We included studies with a cross-sectional study design (S) to enable the identification of relevant keywords and construct comprehensive search strategies for the literature search.

### Inclusion criteria

This review is based on the Population, Intervention, Comparator, and Outcome (PICO) framework. It focuses on women of reproductive age, female adolescents, or the general population (P) and their utilization of essential maternal health services (I), comparing it to the absence of such services (C). The outcome of interest is the impact of COVID-19 on essential depression, anxiety, and maternal health services (O). The studies included were peer reviewed or preprint or grey literature which the outcome assess health service or health impact like depression, anxiety or stress in Ethiopia. The articles had to be written and published in the English language between February 2020 and February 20, 2024.

### Exclusion criteria

The research excluded studies that focused solely on service adaptation and mitigation strategies without providing data on health services utilization or health impact. Additionally, letters, case reports and series, editorial reports, commentaries, reviews, and guidelines were also excluded from the study.

### Data sources and search strategy

A thorough literature search was conducted from February 20, 2020, to February 20, 2024, using various reputable databases such as PubMed/Medline, Science Direct, Cochrane Library, Web of Science, Scopus, and Google Scholar. The search utilized Medical Subject Headings (MeSH) and included the following terms: “impact”, “effect”, “influence”, “COVID-19”, “SARS-CoV-2”, “depression”, “anxiety”, “stress”, “coronavirus”, “novel coronavirus”, “coronavirus disease 2019”, “maternal health service”, “maternal care service”, “health service utilization”, “health care utilization”, “family planning service”, “family planning use”, “antenatal care”, “prenatal care”, “skilled birth attendant”, “institutional delivery”, “health facility delivery”, “postnatal care”, “postpartum care”, “abortion care”, “abortion service”, and “Ethiopia”. The search strategies incorporated a combination of Boolean operators (AND, OR) and truncation. Additionally, the reference lists of relevant studies were manually reviewed to identify any articles that may have been missed during the electronic search.

### Data extraction

The process of extracting data involved using a Microsoft Excel template, which underwent multiple rounds of testing and revision as needed. The authors, who had extensive experience, performed the extraction. The extracted descriptive variables included a wide range of aspects, such as the region where the study was conducted, the study design, the study period, the study setting, the data collection method, the sample size, and the impact of COVID-19 on the outcome.

### Study data management

After conducting a thorough search and collecting various articles, we proceeded to remove any duplicate files. This screening process consisted of two stages: initially evaluating the titles and abstracts, and then conducting a full-text screening. To ensure accuracy, two independent authors used the EndNote software to assess the potential relevance of each article for further review. The assessment was based on a predefined set of criteria for inclusion and exclusion. In cases where there were differences in the reviewers’ assessments, they were resolved through discussion and by seeking input from a third reviewer. To maintain an audit trail, electronic records were kept for both the included and excluded studies, with clear explanations provided for any exclusions made.

### Quality assessment and risk of bias

To assess the risk of bias in the study, a quality assessment checklist for prevalence studies was employed. This checklist, developed by Hoy and colleagues, consists of nine items that are crucial in evaluating the quality of a study (24). These items include the target population, sampling frame, sampling method, response rate, data collection procedures, study case definition, study instruments, and parameters for the numerator and denominator. Each item contributes to a total score of 9. Based on the scores obtained, the studies were categorized as having a low-risk (0–3), moderate-risk (4–6), or high-risk (7–9) of bias. Each study underwent an independent evaluation, and the majority of them demonstrated a low risk of bias. To ensure the reliability of the results, studies with a high risk of bias were excluded from the final analysis.

### Sensitivity analyses

A thorough sensitivity analysis was conducted to assess how individual studies affected the overall estimation of prevalence. Each study was methodically removed, and the resulting impact on the estimate was carefully examined. Surprisingly, the exclusion of any single study did not have a significant effect on the pooled prevalence estimate. Furthermore, none of the studies fell outside the confidence interval’s lower and upper boundaries. These findings indicate that the collective results of the studies remained strong and consistent, reinforcing the reliability of the overall prevalence estimate.

### Data synthesis and analysis

The data obtained from individual articles was processed using Microsoft Excel 2013 and then exported to R software version 4.2 for further analysis. We conducted random-effects meta-analyses in R software to determine the proportion of COVID-19 impact based on individual studies. This allowed us to estimate the pooled prevalence, along with 95% confidence intervals (C.I.s). To compare cases and controls while accounting for confounding factors, we used R software version 4.2 to estimate the odds ratios (O.R.s). We considered a *p*-value less than 0.05 as statistically significant. To assess the level of heterogeneity, we employed a random-effects model and utilized I^2^ statistics. Specifically, I^2^ values of 25, 50, and 75% represented low, medium, and high heterogeneity, respectively. We also examined the distribution of studies in a funnel plot to assess publication bias. Deviation from a symmetrical funnel shape can indicate the presence of publication bias. Additionally, we assessed the quality control of the study.

The Cochran’s Q test was used to test for heterogeneity, with I^2^ statistics indicating low (25%), moderate (25–50%), and high (>50%) heterogeneity. We also estimated pooled odds ratios for factors associated with long COVID-19 sequelae, considering statistical significance at a *p*-value of less than 0.05 of the I^2^. An explanatory variable was included if data was available from at least two of the studies. Furthermore, we performed subgroup analysis based on potential sources of heterogeneity. In addition, we conducted leave-one-out sensitivity analysis to assess the influence of individual studies on the overall effect, which was presented in tables and figures. Sensitivity and publication bias were also assessed in this systematic review and meta-analysis.

## Results

### Study selection and requirement

**Figure 1:**
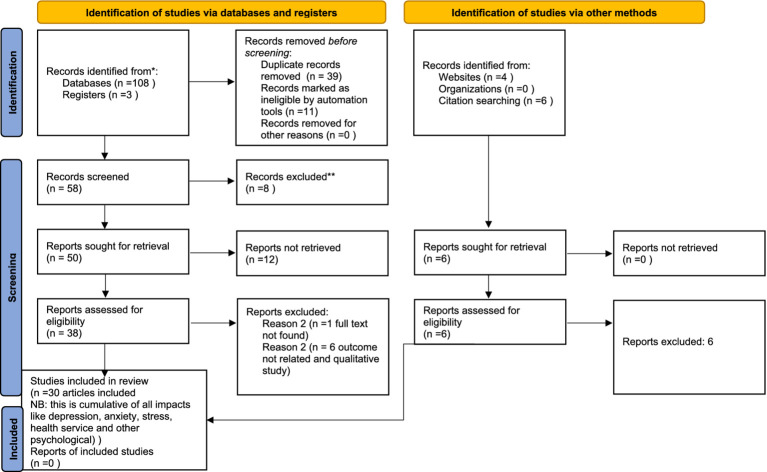
PRISMA flow chart shows study selection for systematic review and meta-analysis of socioeconomic inequalities for prenatal HIV testing in Ethiopia, ^**^protocol, review, unrelated and unclear outcome.

A comprehensive search was conducted using various electronic databases and web-based sources, including reliable databases like Web of Science, PubMed, Embase, Google Scholar, and university repositories. The aim was to identify potential records related to the impacts of COVID-19. Initially, 108 records were identified. To remove duplicate records, EndNote version 8 was utilized, resulting in the removal of 39 duplicates. An automatic tool was then employed to eliminate 28 additional records that did not meet the eligibility criteria. This left us with 11 records for further assessment. Upon closer examination, it was determined that 20 articles were irrelevant as they did not align with the study’s protocol, were review articles, or had unclear outcomes. Consequently, these articles were removed from the library, leaving us with 38 articles. These remaining articles underwent a thorough screening based on their full text. During the full-text screening, four articles were found to be unsuitable for inclusion and were therefore removed. Finally, 30 articles were reviewed for this systematic review and meta-analysis. For a visual representation of these results, please refer to Figure 1. All included studies were cross sectional and studied in all regions of the country Ethiopia. The studies included were 15 articles for depression and anxiety, 11 articles for stress and 7 articles were included for psychological problems.

### Pooled estimate of impacts

#### Anxiety

**Figure 2:**
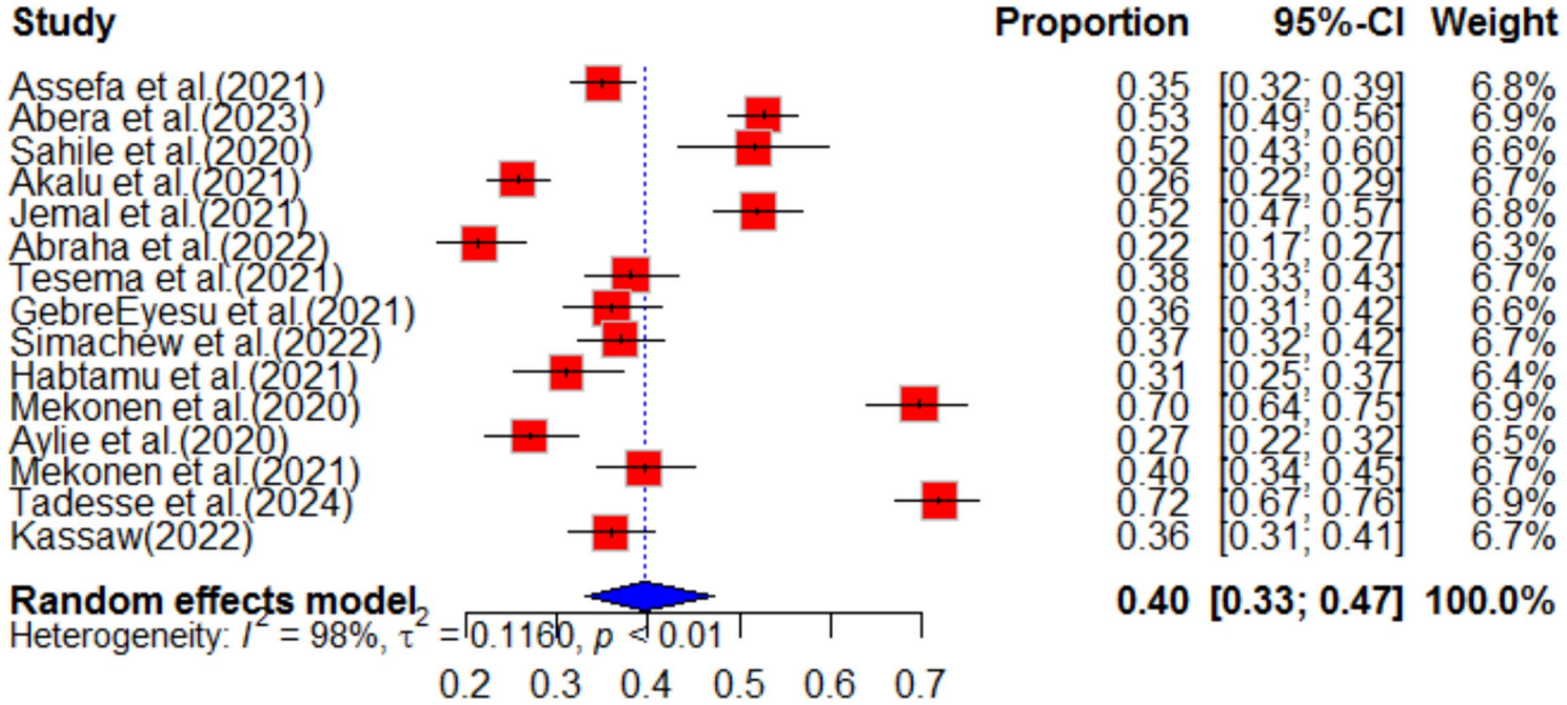
Forest plot of anxiety as impact of COVID-19 in Ethiopia.

**Figure 3:**
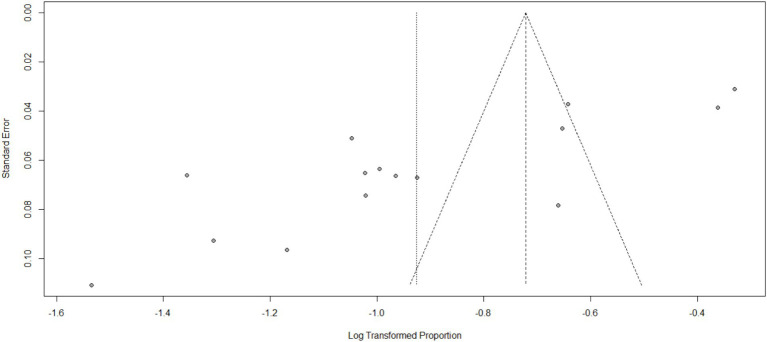
Funnel plot for anxiety as impact of COVID-19 in Ethiopia.

**Figure 4:**
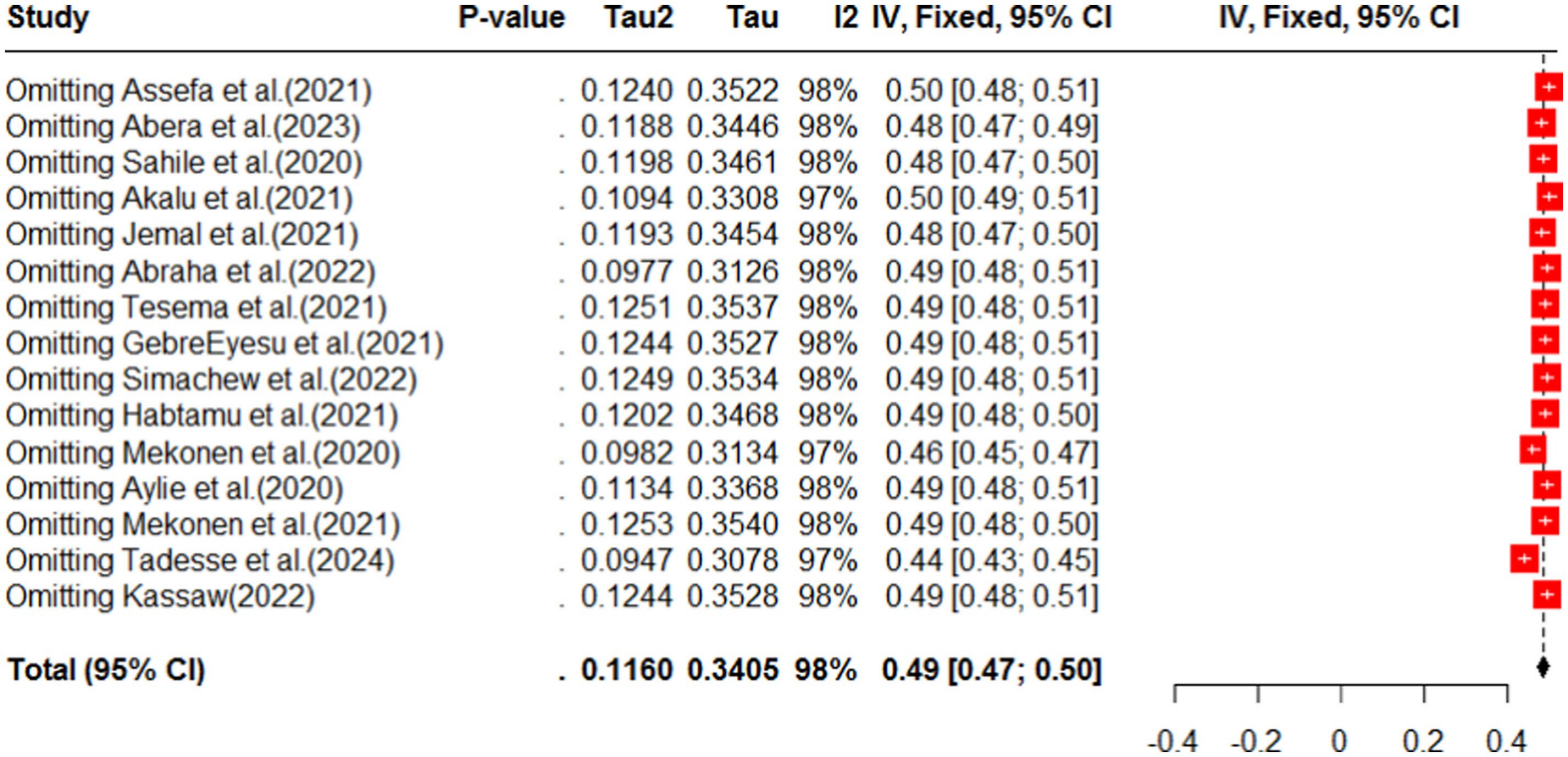
Sensitivity analysis for omitting a study in anxiety as impact of COVID-19 in Ethiopia.

The impact of COVID-19 on mental health has been widely studied, and the most common effects reported are anxiety, depression, stress, and psychological distress. Additionally, COVID-19 has had an impact on maternal health services, including family planning, institutional delivery, safe abortion, postnatal care, and antenatal care. Specifically regarding anxiety, a pooled analysis of multiple studies found that the prevalence of anxiety related to COVID-19 was estimated to be 40% (95%CI: 33–47), with a high level of heterogeneity (98%) and a significant *p*-value (<0.01). Due to the high heterogeneity, a random effect model was used for the analysis (as shown in Figure 2). The funnel plot for anxiety indicated some asymmetry (as shown in Figure 3), suggesting potential publication bias. To address this, a sensitivity analysis was conducted by omitting one study, which revealed that four studies deviated from the center. To balance the findings, a trim-and-fill analysis was performed by adding seven studies Figure 4.

**Figure 5:**
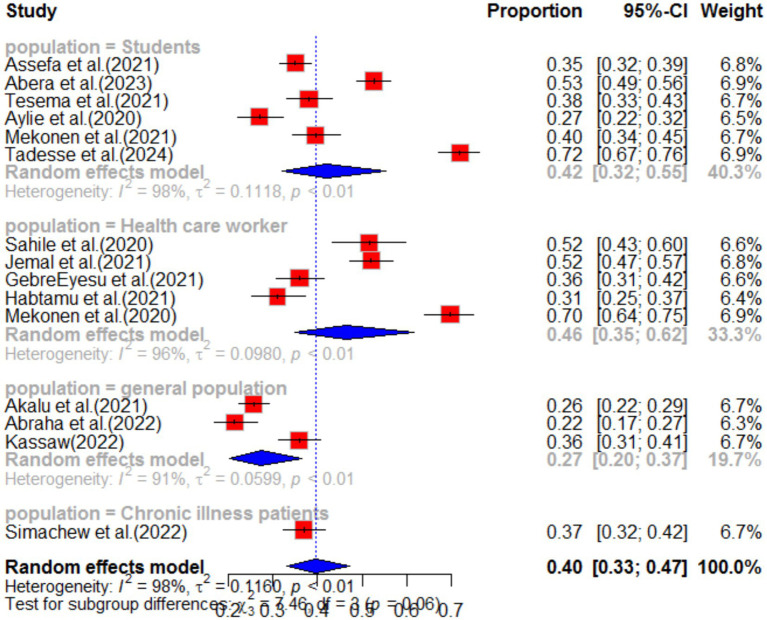
Sub-group analysis by population type of anxiety as impact of COVID-19 in Ethiopia.

**Figure 6:**
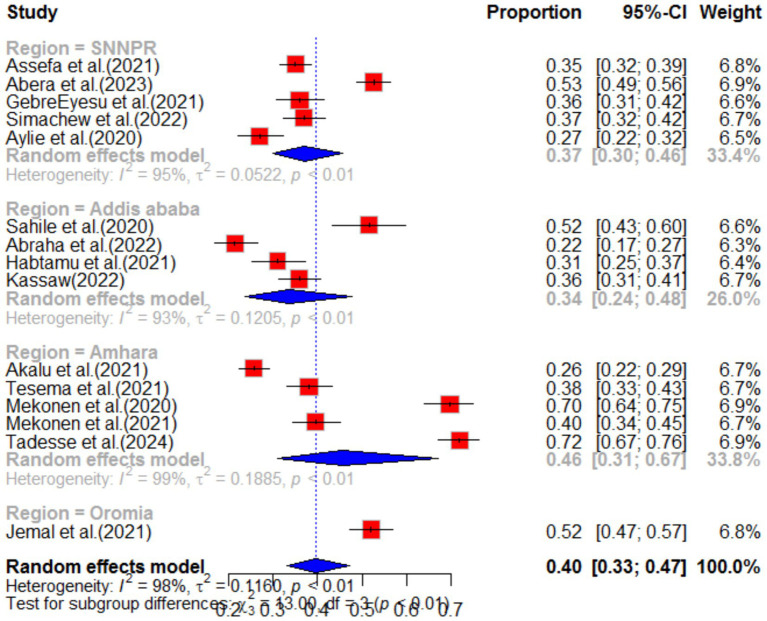
Sub-group analysis by region of anxiety as impact of COVID-19 in Ethiopia.

Subgroup analysis was conducted based on region and population type in the primary study. The highest pooled prevalence of anxiety was observed among healthcare workers, with a prevalence of 46% (95%CI: 35–62), and a high level of heterogeneity (I^2^ = 96%, *p*-value<0.01). The second highest prevalence of anxiety was observed among students, with a prevalence of 42% (95%CI: 32–55) (as shown in Figure 5). When analyzing anxiety based on region, the highest prevalence was observed in the Amhara region (46, 95%CI: 31–67), followed by the South Nation Nationality region (37, 95%CI: 30–46) (as shown in Figure 6). Furthermore, the effect size of low income on anxiety was found to be 2.23 times more likely (95%CI: 1.53–3.25) compared to high-income individuals. This analysis used a fixed effect model due to low heterogeneity (I^2^ = 16%, *p*-value = 0.27) (Table 1).

**Table 1:** Maternal health service and mental health impact of COVID-19 in Ethiopia.

Impact	Pooled impact (95%CI)	Factors	Pooled odds ratio	Heterogeneity	Model used
Anxiety	40 (33–47)	Age above 25 years	2.99 (0.73–12.30)	I^2^ = 63%, *p* value = 0.10	Random effect
		Low income	2.23 (1.53–3.25)	I^2^ = 16%, *p* value = 0.27	Fixed effect
Depression	41 (34–50)	Female sex	2.25 (1.69–3.00)	I^2^ = 0%, *p* value = 0.87	Fixed effect
		Living in prison	5.87 (2.53–13.65)	I^2^ = 0%, *p* value = 0.98	Fixed effect
		Married	3.64 (1.85–7.19)	I^2^ = 58%, *p* value = 0.09	Random effect
		Working in emergency department	2.17 (1.32–3.57)	I^2^ = 0%, *p* value = 0.90	Fixed effect
Psychological	41 (27–62)	Female sex	1.76 (1.42–2.18)	I^2^ = 0%, *p* value = 0.81	Fixed effect
		No social support	3.72 (2.15–6.44)	I^2^ = 0%, *p* value = 0.86	Fixed effect
Family planning	11 (2–62)			I^2^ = 100, *p* value<0.01	Random effect
Institutional delivery	16 (10–26)			I^2^ = 99, *p* value<0.01	Random effect
Postnatal care	25 (8–83)			I^2^ = 99, *p* value<0.01	Random effect
Antenatal care	5 (4–5)			I^2^ = 0, *p* value = 0.55	Fixed effect
Safe abortion	14 (10–18)			I^2^ = 80, *p* value = 0.03	Random effect

Overall, these findings highlight the significant impact of COVID-19 on mental health, particularly in terms of anxiety. It is important to address these issues and provide appropriate support and interventions, especially for high-risk groups such as healthcare workers and students.

#### Depression

**Figure 7:**
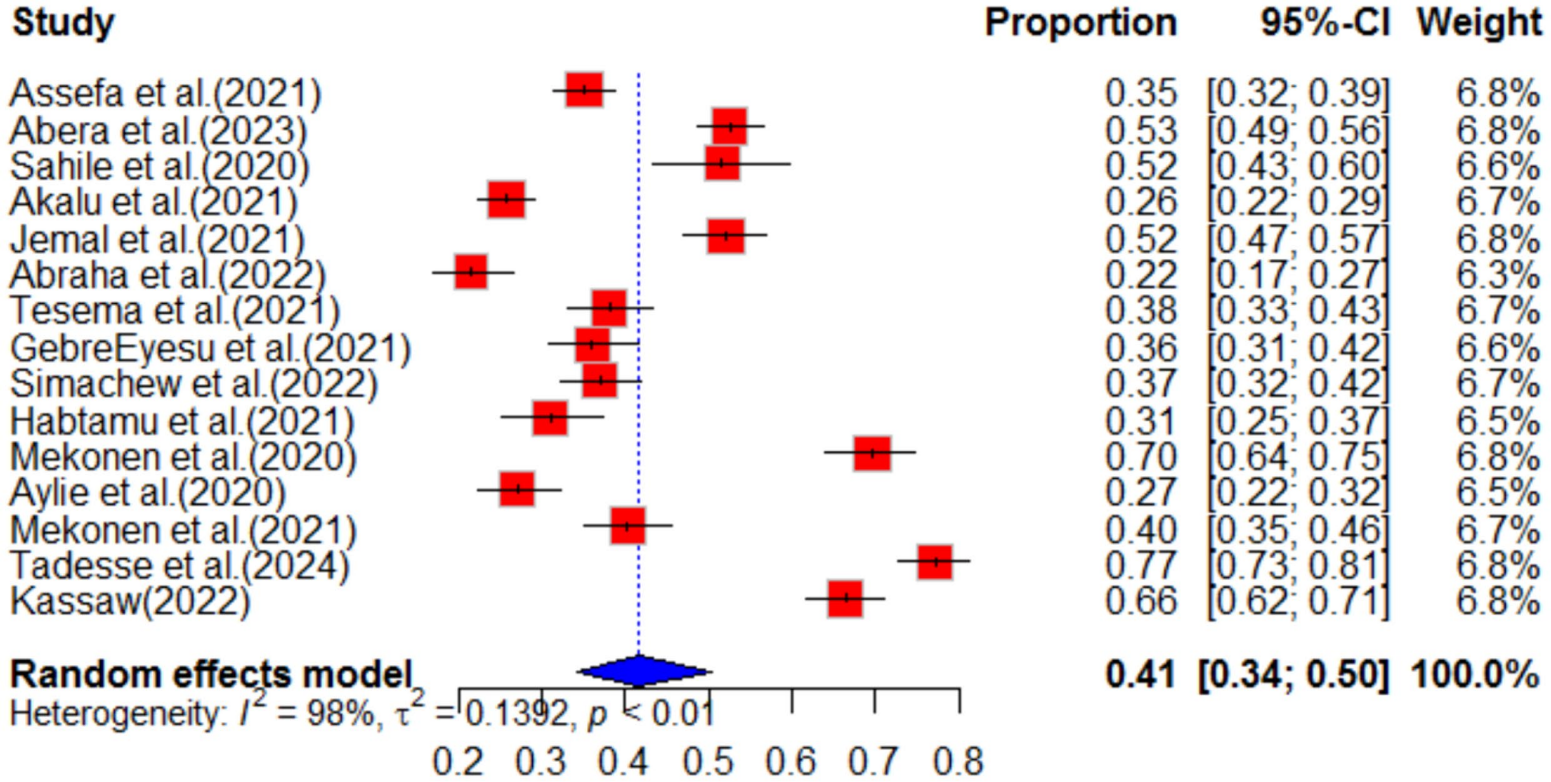
Forest plot for depression as impact of COVID-19 in Ethiopia.

**Figure 8:**
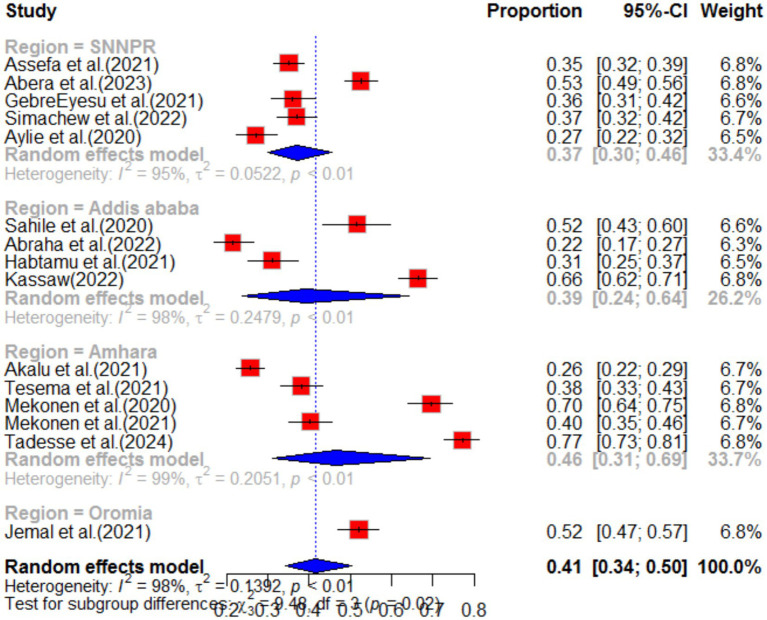
Sub-group analysis for depression by region.

**Figure 9:**
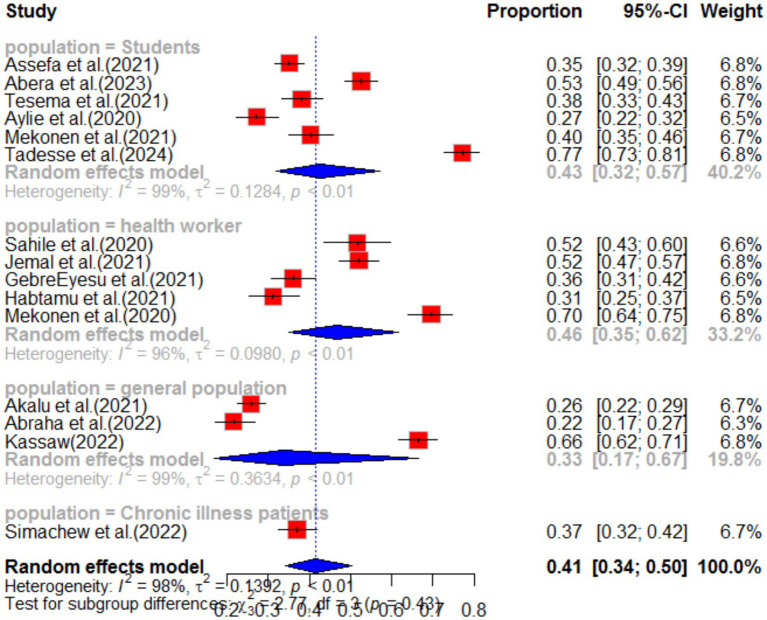
Sub-group analysis for depression by population type.

**Figure 10:**
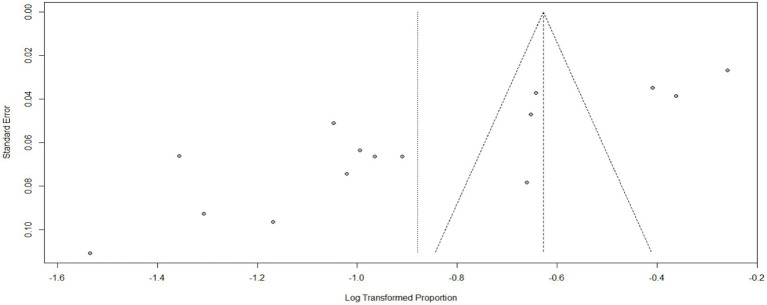
Funnel plot for depression as impact of COVID-19 in Ethiopia.

The impact of COVID-19 on depression was assessed in 15 articles. The systematic review found that the pooled prevalence of depression was 41% (95%CI: 34–50), with high heterogeneity (I^2^ = 98%, *p*-value <0.01). Sub-group analysis revealed that the highest pooled depression effect was observed in the Amhara region 46% (95%CI: 31–69) and among health workers 46% (95%CI: 35–62), as shown in Figures 7, 8, respectively. The funnel plot indicated asymmetry in the studies, as depicted in Figures 9, 10. A sensitivity analysis was conducted by omitting one study, but it showed no significant variation from the pooled results.

The systematic review also examined the effect of different factors on depression. Based on the findings, females were 2.25 times more likely (POR = 2.25, 95%CI 1.69–3.00) to experience depression due to COVID-19 compared to males. Living in prison increased the likelihood of experiencing depression by 5.87 times (POR = 5.87, 95%CI 2.53–13.65) compared to the general population. Marital status also had an effect, with married individuals being 3.64 times (POR = 3.64, 95%CI 1.85–7.19) more likely to experience depression compared to unmarried individuals. Additionally, working in an emergency department was associated with a higher risk of depression due to COVID-19. Health workers in the emergency department were 2.17 times (POR = 2.17, 95%CI 1.32–3.57) more likely to experience depression compared to those in other departments (see Table 1).

#### Stress

**Figure 11:**
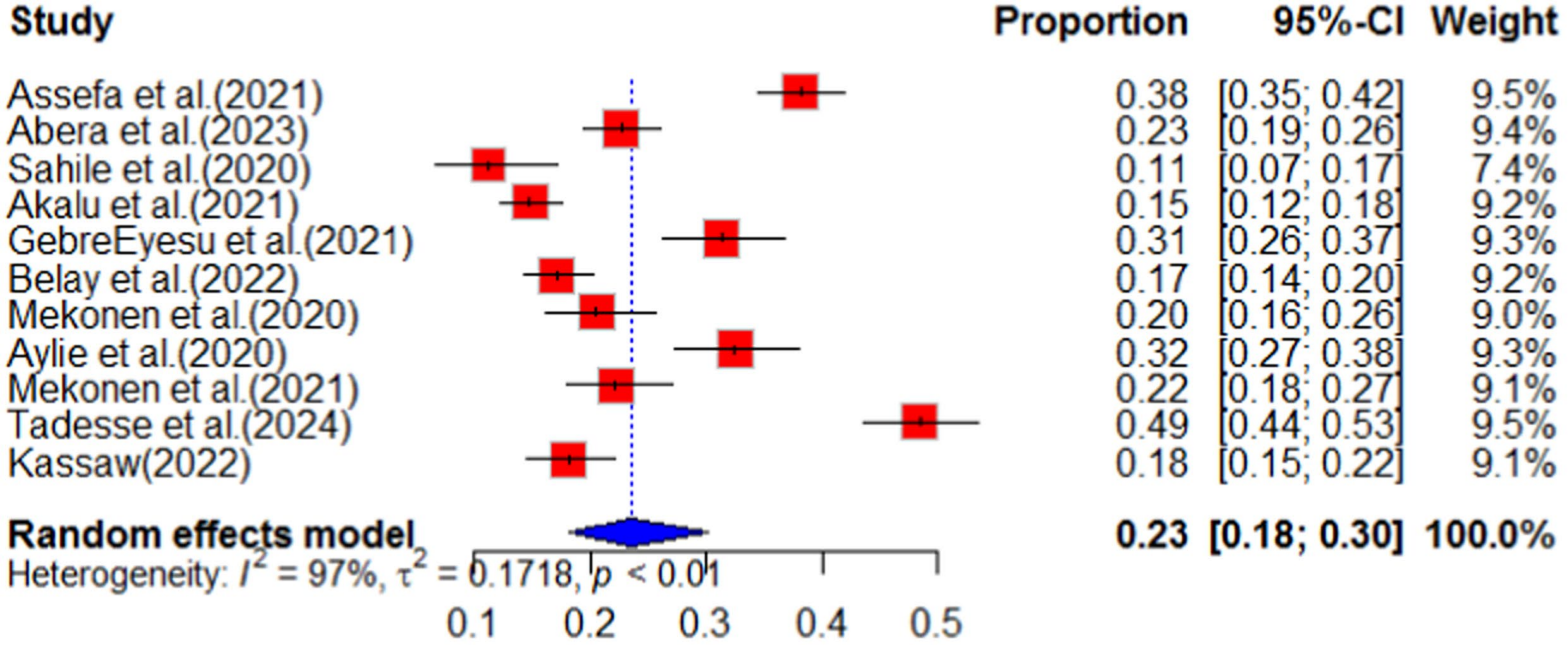
Forest plot for stress as impact of COVID-19 in Ethiopia.

**Figure 12:**
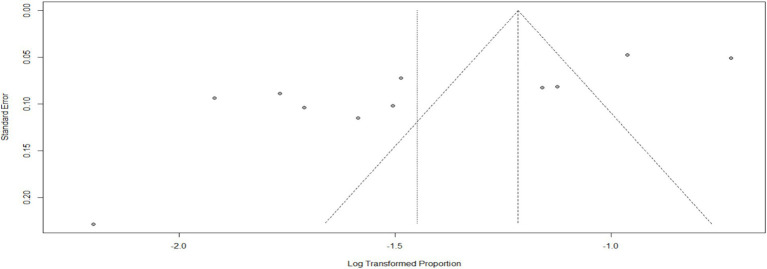
Funnel plot for stress as impact of COVID-19. *N* = 22 (adding 7 articles).

**Figure 13:**
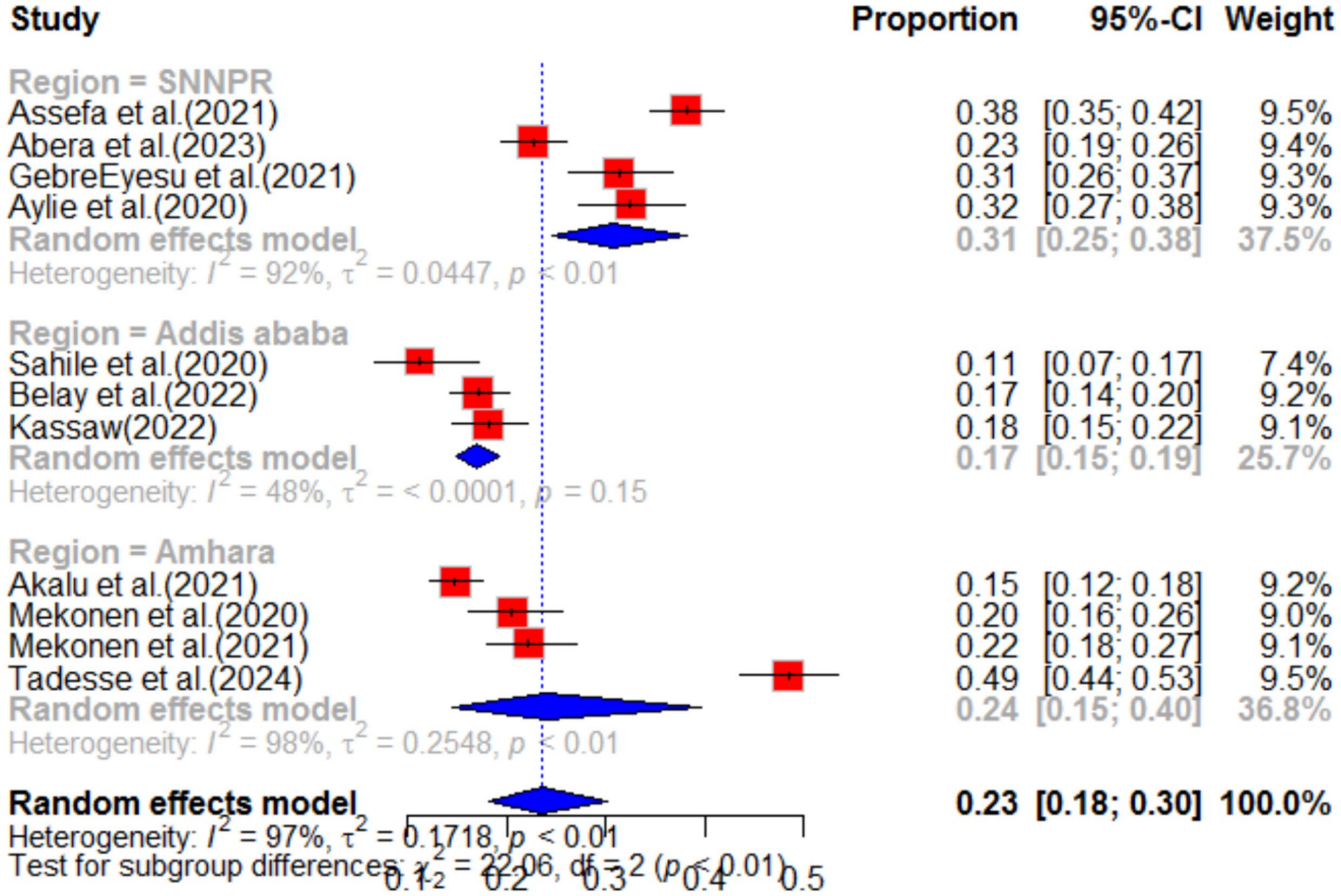
Sub-group analysis by region of stress as impact of COVID-19 in Ethiopia.

According to a systematic review and meta-analysis conducted in Ethiopia, the study included a total of 11 articles. The pooled prevalence of stress among the population was found to be 23% (95%CI: 18–30), with a high level of heterogeneity (I^2^ = 97%) and a significant *p*-value of less than 0.01 (Figure 11). The funnel plot analysis revealed asymmetry in the distribution of studies, indicating potential publication bias (Figure 12). However, the study’s meta-bias analysis indicated that there was no bias related to sample size or publication year. In the sub-group analysis, it was observed that the highest pooled prevalence of stress was found in the SNNPR region, with a prevalence of 31% (95%CI: 25–38). Similar to the overall analysis, this subgroup also exhibited high heterogeneity (I^2^ = 92%) and a significant *p*-value of less than 0.01 (Figure 13). These findings suggest that stress related to the COVID-19 pandemic is prevalent in Ethiopia, particularly in the SNNPR region. However, it is important to consider the limitations of the included studies, such as potential bias and heterogeneity, when interpreting these results.

#### Psychological

**Figure 14:**
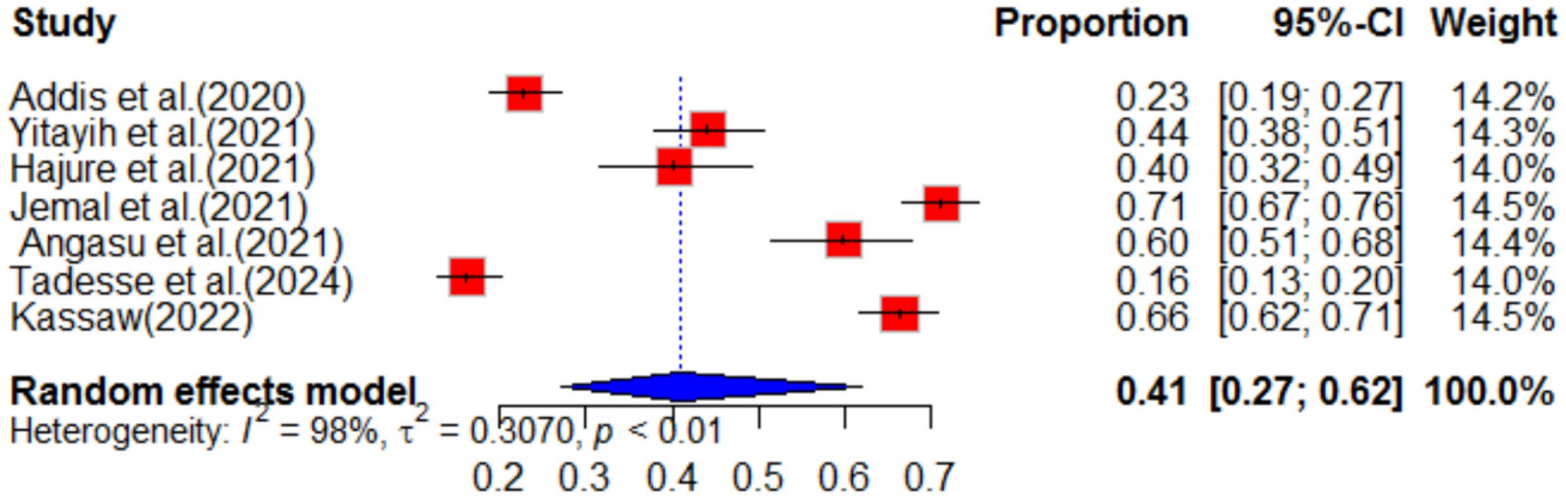
Forest plot for psychological impact of COVID-19 in Ethiopia.

This study conducted a systematic review of the psychological impact of COVID-19, with a particular focus on its effects in Ethiopia. The review analyzed seven primary studies and found that the pooled impact of COVID-19 on psychology was 41% (95% CI: 27–62), with a high degree of heterogeneity (I^2^ = 98%, *p* value<0.01) (as shown in Figure 14).

**Figure 15:**
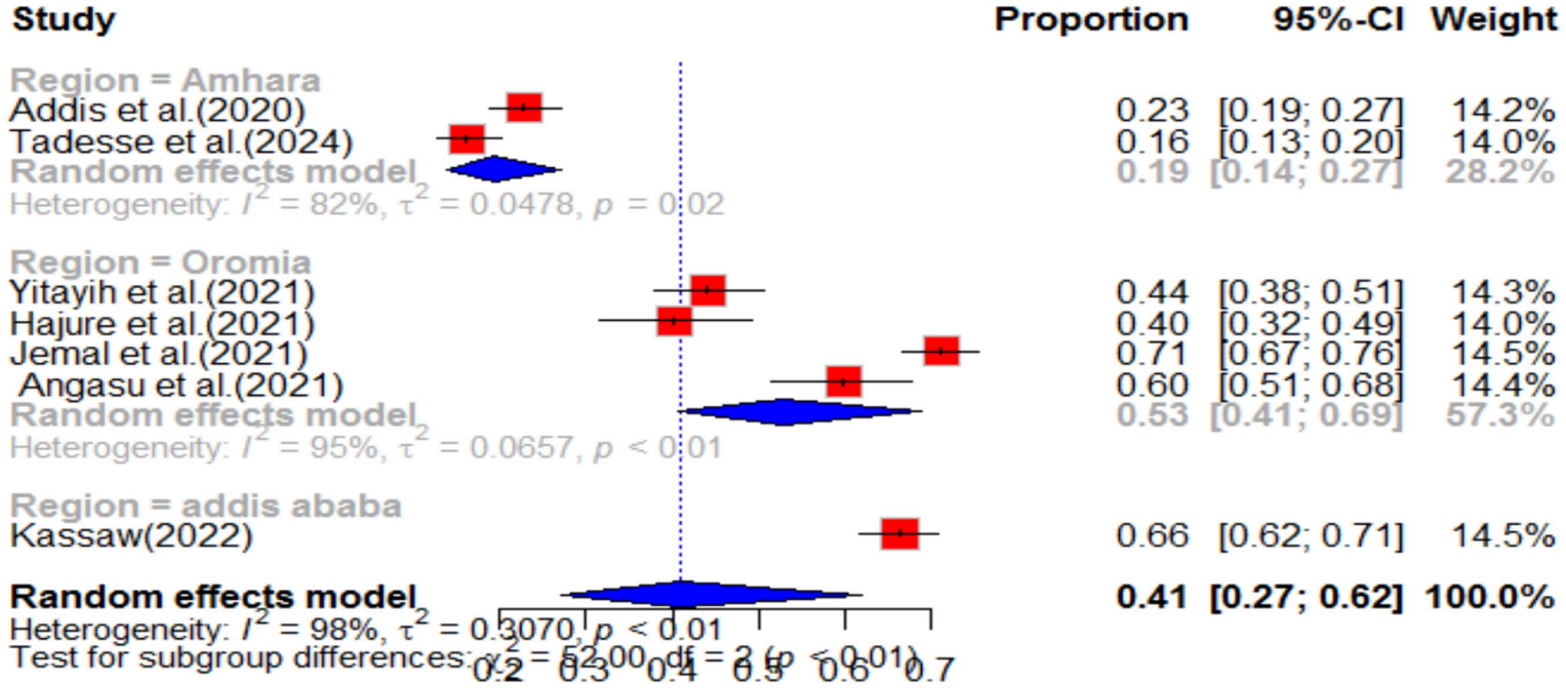
Forest plot for sub-group analysis by region for stress as impact of COVID-19 in Ethiopia.

Sub-group analysis revealed that the Oromia region in Ethiopia experienced the highest psychological impact at 53% (95% CI: 41–69) (Figure 15). The review also analyzed the effect of different variables on the psychological impact of COVID-19. It found that females were 76% more likely (POR = 1.76, 95% CI: 1.42–2.18) to experience psychological impact compared to males, with no heterogeneity (I^2^ = 0%, *p*-value = 0.81). Additionally, the review found that individuals who lacked social support during the pandemic were 3.72 times (95% CI: 2.15–6.44) more likely to experience psychological impact, with no heterogeneity (I^2^ = 0%, *p*-value = 0.87) (as shown in Table 1). Overall, this study highlights the significant psychological impact of COVID-19 and the importance of social support in mitigating its effects.

## Impact of COVID-19 on health services

The impact of COVID-19 on maternal health services has been assessed by examining the pooled prevalence of reductions in these services. The findings indicate that antenatal care (ANC), institutional delivery, postnatal care (PNC), safe abortion, and family planning have all been affected. Among these services, PNC, institutional delivery, family planning, and safe abortion have experienced the highest impact due to COVID-19. Specifically, in the country under study, COVID-19 has resulted in a 25% reduction (95%CI 8–83) in postnatal care services. Similarly, institutional delivery has seen a reduction of 16% (95%CI 10–26) compared to the pre-pandemic scenario. Safe abortion services have also been significantly affected, with a 14% reduction (95%CI 10–18) in Ethiopia. Furthermore, institutional delivery during the pandemic has experienced an 11% reduction in service utilization. These impacts of COVID-19 on maternal health services are summarized in Table 1. It is important to note that ANC services in Ethiopia have also been affected, with a 5% reduction attributed to COVID-19. These findings highlight the considerable impact of COVID-19 on maternal health services, emphasizing the need for appropriate measures to mitigate these effects and ensure the continued provision of essential care to pregnant individuals.

## Discussion

This study aimed to assess the impact of COVID-19 on mental health and maternal health services in Ethiopia. Our review explores the effects of COVID-19 on mental health and maternal health, as well as the factors associated with these impacts in Ethiopia. We found that the pandemic has significantly affected psychiatric outcomes and maternal health services, with a noticeable reduction compared to pre-pandemic times. The prevalence rates varied among different studies, which could be attributed to differences in measurement scales, reporting patterns, and cultural factors (25–27). Regional disparities were also observed in terms of the psychological well-being of the general public during the outbreak. These differences can be attributed to variations in the severity of the outbreak, the national economy, government preparedness, availability of medical resources, and the effective dissemination of COVID-related information. Furthermore, the stage of the outbreak in each region also influenced the psychological responses of the public. At the beginning of the outbreak, individuals faced challenges such as mandatory quarantine, unexpected unemployment, and uncertainty, which led to a higher prevalence of adverse psychological symptoms (27, 28). When evaluating the psychological impact of the coronavirus outbreak, it is crucial to consider the duration of psychiatric symptoms. Acute psychological responses to stressful or traumatic events can sometimes serve as protective mechanisms and have evolutionary significance (29).

This systematic review examined the impact of COVID-19 on anxiety in Ethiopia, drawing from 50 different articles. Out of these, 15 articles reported anxiety as a consequence of the pandemic in various regions of the country. The pooled prevalence of anxiety as a result of COVID-19 was found to be 40% (95% CI; 33–47). This finding is consistent with other systematic studies, which also reported anxiety as one of the impacts of COVID-19 (30, 31). The study highlights that the pandemic has led to an increase in anxiety among individuals, indicating a rise in mental health problems during this period (32). This implies mental health problem increase during the pandemic. The sub-group analysis revealed that healthcare workers experienced higher levels of anxiety compared to others, with a pooled prevalence of 46% (95% CI; 35–62). This suggests that the work environment has a significant influence on the mental impact of COVID-19 (33). Overall, this study underscores the need for mental health support and interventions for individuals and healthcare workers in particular, to mitigate the negative impact of COVID-19 on mental health.

In our systematic review and meta-analysis, we discovered a significant determinant that affects anxiety levels during the COVID-19 pandemic in Ethiopia. Specifically, we found that age and income play a crucial role in determining anxiety levels. Notably, individuals with lower income levels experienced a more pronounced impact on anxiety. This finding aligns with similar studies conducted in various regions around the world (34–36). It suggests that the COVID-19 pandemic has had a significant impact on resource-limited settings and individuals in third-world countries. Consequently, it is imperative to implement program interventions and economic support measures to effectively mitigate the effects of the pandemic. Numerous reports have indicated that individuals with poor economic status, lower education levels, and unemployment face significant risk factors for experiencing symptoms of mental disorders, particularly depressive symptoms, during the ongoing pandemic. The global outbreak of the coronavirus has necessitated strict stay-at-home orders and has caused a decline in the demand for services and goods, which has had a detrimental impact on local businesses and industries worldwide (37–40). The decrease in quality of life and the uncertainty resulting from financial hardships can further increase the vulnerability of individuals to developing adverse psychological symptoms (41).

The COVID-19 pandemic has had a significant impact on mental health, particularly in terms of depression. A systematic review was conducted, analyzing fifty primary articles from various regions. The pooled prevalence of depression was found to be 41% (95% CI: 34–50), with the highest prevalence observed in the Amhara region at 46% (95% CI: 31–69). These findings are consistent with other studies conducted worldwide (27, 30, 42–45), suggesting that depression may be one of the most common impacts of COVID-19 post-pandemic. The review also identified several sociodemographic factors that contribute to depression in the context of COVID-19. Being female, married, or living in prison were found to increase the likelihood of experiencing depression. Specifically, females were 2.25 times more likely to experience depression compared to males. This aligns with previous research (43, 44), which suggests that women may be more affected due to their higher representation in industries negatively impacted by the pandemic, such as retail, service, and healthcare. Additionally, women may exhibit differential neurobiological responses to stressors, potentially contributing to higher rates of mental disorders.

Living in prison was also found to significantly increase the risk of depression, with individuals in prison being 5.87 times more likely to experience depression compared to those living in their own homes. This finding is consistent with previous studies and highlights the need to consider the mental health implications for incarcerated individuals when designing programs and policies. Marital status was another factor identified in the review, with individuals who were married being 3.64 times more likely to experience depression compared to their counterparts. This finding aligns with previous research conducted in different parts of the world, emphasizing the importance of considering sociodemographic characteristics in program and policy design.

Furthermore, working in the emergency department was found to increase the impact of COVID-19 on mental health. Health workers in this setting were 2.17 times more likely to experience depression during the pandemic. This finding is consistent with previous studies and underscores the need to address the mental well-being of healthcare professionals in emergency settings.

Overall, this systematic review provides valuable insights into the impact of COVID-19 on depression and identifies various factors that contribute to its occurrence. These findings have important implications for the development of targeted interventions and policies aimed at mitigating the mental health effects of the pandemic.

Moreover, stress has been identified as one of the significant consequences of the COVID-19 pandemic in Ethiopia. A comprehensive analysis comprising 11 articles was conducted to evaluate the impact of COVID-19 in Ethiopia. The pooled analysis of this systematic review and meta-analysis revealed that the prevalence of stress was estimated to be 23% (95% confidence interval: 18–30). This finding aligns with previous studies (27, 44, 45) conducted in various regions across the globe, indicating a similar impact of the pandemic on stress levels. The impact of COVID-19 is not only evident in terms of physical health but also in psychological and maternal health services. A systematic review was conducted, analyzing seven articles from various regions of different countries. The findings revealed that in Ethiopia, the pooled prevalence of psychological impact due to COVID-19 was 41%. Furthermore, the subgroup analysis indicated that Oromia had the highest prevalence of psychological impact among the regions in the country. These findings were consistent with studies (44) conducted in other parts of the world, suggesting that COVID-19 significantly affects the mental well-being of individuals and communities. The systematic synthesis of evidence also explored the sociodemographic factors contributing to the psychological impact of COVID-19 in Ethiopia. The analysis identified that being female and lacking social support were significant factors. These findings align with studies conducted in different areas worldwide, highlighting the disruption of sociocultural bonds within communities caused by the pandemic. Therefore, it is crucial for pandemic control and impact mitigation strategies to consider the social structure and sociodemographic situation of the affected communities.

As the COVID-19 pandemic continues to affect individuals worldwide, those with pre-existing chronic conditions are particularly vulnerable and may experience heightened anxiety and distress. This is due to their compromised immunity, which makes them more susceptible to the virus and increases their risk of mortality. For example, patients with systemic lupus erythematosus may face significant health challenges in the face of COVID-19. Reports have also indicated that individuals with common chronic conditions such as diabetes, hypertension, and coronary heart disease may experience a higher death rate if infected with the virus. However, the exact causes of this phenomenon remain unknown, leaving those with these conditions feeling uncertain and afraid. In addition to the health risks posed by COVID-19, patients with pre-existing conditions may also face practical concerns such as postponement or inaccessibility of medical services and treatment. As COVID-19 affect patients, preventative care for other diseases may unintentionally, increasingly utilize hospitals and medical resources. Furthermore, individuals with a history of mental disorders or current diagnoses of psychiatric illnesses may be particularly sensitive to the external stressors associated with the pandemic, such as social isolation. It is important to recognize and address the unique challenges faced by individuals with pre-existing conditions during this difficult time (28, 46–48).

The maternal health services are also one of the services impacted by COVID-19 during the pandemic. The maternal health service reduction was observed in this systematic review. The maternal health services reduced due to COVID-19 were family planning, safe abortion, institutional delivery, PNC, and ANC. The comprehensive review highlights the significant decrease in healthcare utilization worldwide, emphasizing the need to prioritize addressing the unmet needs of individuals with non-COVID-19 illnesses. Primary studies consistently emphasize the importance of monitoring the long-term consequences of missed care, launching public campaigns to encourage timely medical attention, and enhancing preparedness to minimize future instances of missed care during subsequent waves of the pandemic. The urgent calls to action are further reinforced by evidence of excess population mortality, including deaths unrelated to COVID-19, as well as notable increases in out-of-hospital cardiac arrests and emergency phone line contacts (49, 50). On the other hand, the review also reveals that reductions in healthcare utilization were often more pronounced for less severe or milder conditions. This, combined with existing evidence on the issue of overmedicalization, suggests that for some individuals, missing care may not have resulted in harm (51, 52). This unprecedented natural experiment, induced by the pandemic and its impact on healthcare utilization, presents a valuable opportunity to gain insights into the services that populations and healthcare systems considered of lesser priority when reallocating resources to essential services was crucial for minimizing mortality during a crisis. As previously suggested, the significant decrease in non-urgent visits (53, 54) to emergency departments worldwide indicates an opportunity to develop and implement new strategies and care models that optimize the appropriateness of such visits in the future.

## Conclusion

This systematic review focused on the impact of the COVID-19 pandemic on the psychological well-being of the general public. It highlighted the high prevalence of adverse psychiatric symptoms and emphasized the need to prioritize mental health alongside efforts to control the spread of the virus. The review also identified sociodemographic factors such as age, sex, low income level, marital status, and living conditions as significant predictors of mental health impacts.

Furthermore, the review highlighted the reduction in maternal health services in Ethiopia due to the pandemic. It stressed the importance of collecting high-quality data and conducting long-term cohort studies to better understand the ongoing changes in healthcare utilization and assess the impacts on health, costs, and equity. The experiences of individuals affected by the pandemic, particularly the most vulnerable populations, should be taken into account when designing interventions and providing support.

For program designers, the identified determinants should be considered to reduce the impact of COVID-19 in the country. The Ministry of Health in Ethiopia and the health sector should give great emphasis to individuals from low-income levels, females, and all other predictors identified in this systematic review and meta-analysis. Additionally, rigorous qualitative research should be conducted to understand people’s experiences of avoiding or missing healthcare and to assess professionals’ responses to changes in processes and practices. Unfortunately, no studies were found that specifically examined changes in the utilization of low-value healthcare services during the pandemic.

Overall, this systematic review provides essential baseline data for policymakers and program implementers, highlighting the urgent need to address the mental health challenges posed by the COVID-19 pandemic and to integrate viral risk mitigation with measures to support mental well-being.

## Strength and weakness of the study

This systematic review was conducted at the country level and yielded highly credible findings. To ensure comprehensive coverage, multiple databases were searched manually and electronically for articles suitable for meta-analysis. The information was uniformly abstracted using a predetermined and pretested standard format by two independent reviewers, which helped minimize errors. The meta-analysis included studies from various regions of the country, examining the impacts of COVID-19 during the pandemic, lockdown, and post-lockdown periods. This extensive review and meta-analysis encompassed the period from the onset of COVID-19 until the recent years of 2024, providing robust evidence for program development and policy-making.

Despite its strengths, the review had some potential limitations. Firstly, all the included studies were cross-sectional and written in English, limiting generalizability. Moreover, there was substantial heterogeneity among the studies. Additionally, since the studies relied on self-reported data, there is a possibility of overestimation or underestimation of the prevalence of COVID-19 due to social desirability bias.

## Data availability statement

The original contributions presented in the study are included in the article/supplementary material, further inquiries can be directed to the corresponding author.

## Author contributions

MA: Conceptualization, Data curation, Formal analysis, Funding acquisition, Investigation, Methodology, Project administration, Resources, Software, Supervision, Validation, Visualization, Writing – original draft, Writing – review & editing. DG: Conceptualization, Formal analysis, Funding acquisition, Project administration, Resources, Software, Writing – original draft, Writing – review & editing. YN: Conceptualization, Formal analysis, Funding acquisition, Methodology, Writing – original draft, Writing – review & editing. AA: Formal analysis, Investigation, Methodology, Validation, Visualization, Writing – original draft, Writing – review & editing. GA: Formal analysis, Funding acquisition, Investigation, Methodology, Software, Visualization, Writing – original draft, Writing – review & editing.
